# Clearing-induced tisssue shrinkage: A novel observation of a thickness size effect

**DOI:** 10.1371/journal.pone.0261417

**Published:** 2021-12-16

**Authors:** R. C. M. Vulders, R. C. van Hoogenhuizen, E. van der Giessen, P. J. van der Zaag

**Affiliations:** 1 Philips Research Laboratories, Eindhoven, The Netherlands; 2 Fontys University of Applied Sciences, Eindhoven, The Netherlands; 3 Zernike Institute for Advanced Materials, University of Groningen, Groningen, The Netherlands; University of Colorado Denver, UNITED STATES

## Abstract

The use of clearing agents has provided new insights in various fields of medical research (developmental biology, neurology) by enabling examination of tissue architecture in 3D. One of the challenges is that clearing agents induce tissue shrinkage and the shrinkage rates reported in the literature are incoherent. Here, we report that for a classical clearing agent, benzyl-alcohol benzyl-benzoate (BABB), the shrinkage decreases significantly with increasing sample size, and present an analytical formula describing this.

## Introduction

Solvent-based tissue clearing is a widely used methodology to render an otherwise opaque sample optically transparent. Using tissue clearing in combination with various optical imaging techniques enables the study of various organs and organisms, such as mouse brains [1–3], larvae and spinal cords [4] as well as tumours including their (micro)environment [5] such that the overall structure and interaction with the neighboring tissue structures can be studied and understood. This has been important for the advancing understanding in neurology and oncology.

Typically in tissue clearing, samples are immersed in various solvents and incubated to achieve complete permeation of the solvent and clearing of the specimen, as demonstrated already over a century ago [6]. Tissue clearing is achieved by consecutive steps of: fixation (by means of paraformaldehyde), dehydration, de-lipidation (using methanol) and finally refractive index matching using a clearing agent (such as mixture of benzyl-alcohol and benzyl-benzoate (BABB)) [4, 7]. Solvent-based clearing strategies all share the disadvantage that during the different processing steps alterations in the overall sample size and possibly architecture may occur, due to shrinkage of the tissue [8]. Alternatives to solvent-based clearing procedures are simple immersion in an aqueous solution with sugars or using hyperhydration [9]. Finally, a third category is hydrogel-based clearing methods, in which biomolecules are covalently linked to an acryl-based hydrogel [3, 10, 11]. Over time, these three basic categories of clearing methods mentioned above have been further developed into various dedicated methods, see for example some of the recently published methods [10, 12–14] and the various recent reviews of clearing methods [9, 11, 15, 16], to which we refer for further reading on the various tissue-clearing methods and their pros and cons as well as the different extent of volume changes that these clearing methods cause. Yet, in view of the high-quality clearing they produce, solvent-based clearing methods based on BABB [1, 4, 17] or derived agents [18] play a prominent role in the field [9]. We use the classical BABB-based clearing method as a model system here, since it has been used in seminal studies [1] and is well suited for clearing small samples [4].

Strikingly, upon detailed examination of the literature about the extent of tissue shrinkage induced by clearing agents, a very confusing picture emerges, with various sources pointing to previous literature, which again refers to others. In the end, most sources point to the work by Ertürk *et al*. who reported that the tissue shrinks isotropically in all dimensions by 21%, resulting in a 51% reduction of the 3D volume [4]. However, one of the more recent paper reports different amounts of shrinkage for different tissues (e.g. 55% for mouse brains, 30% for hippocampus and cortex layers, and 10.7% for a mouse torso) [18]. Given that a number of groups are extending the study of tissue analysis in 3D to that of human biopsies, using various means of optical microscopy yet all relying on tissue clearing [17, 19–21], it becomes important to know the extent of tissue shrinkage caused by the use of clearing agents.

## Material and methods

### a. Sample processing

Sampling was performed using needles with different inner diameters: 300, 550, 800, 900 and 1200 μm (BD, New Jersey, USA). Needles and Syringes (BD, New Jersey, USA) were filled with sterile phosphate buffered saline (PBS, Merck, Darmstadt, Germany) prior to puncturing, this to release the samples more conveniently. The 1.6 mm sampling was performed by using a dermal biopsy puncher (Integra, Miltex, York, Pennsylvania, USA, inner diameter 1.67 mm). Biopsies were taken from frozen samples stored at– 20°C. Upon puncturing, the samples were transferred into homemade solvent resistant containers at room temperature (S1 Fig). Briefly, a dual barrel piston container (Nordson EFD, Bedfordshire, England) was cut and fastened on a coverslip (Menzel-glaser, Thermo Scientific, Waltham, Massachusetts, USA) with a two-component epoxy adhesive (UHU, Bühl/Baden, Germany); upon mounting the adhesive was left overnight to solidify.

**Fig 1:**
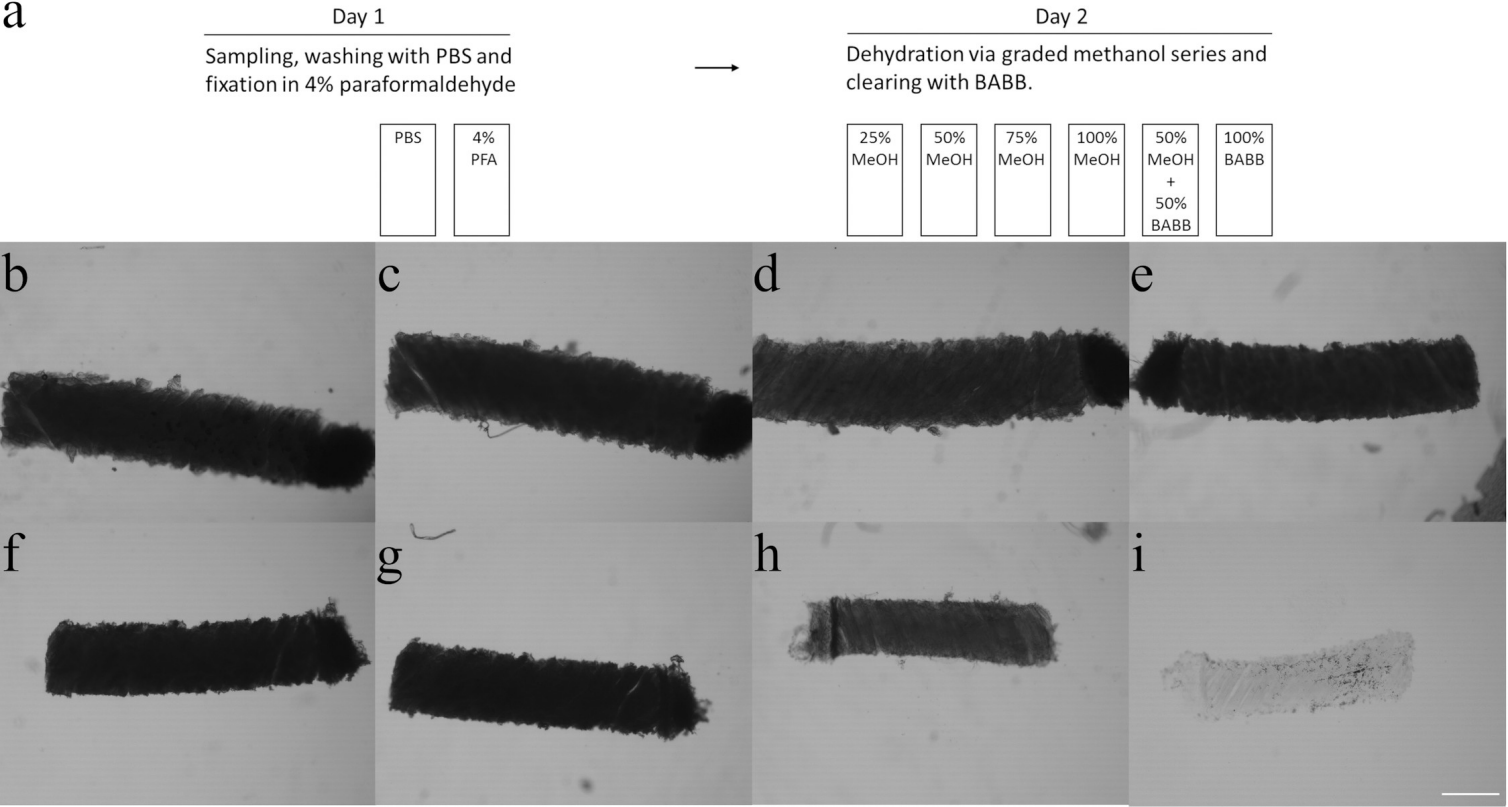
Optical microscopy images acquired after each consecutive process step of the clearing protocol for a rat liver sample. Panel **(a)** gives a schematic representation of the procedure. Panel **(b)** shows the sample immediately after sampling, in **(c)** after overnight fixation, **(d-g)** after the graded methanol series, **(h)** after the 50% methanol / 50% BABB step and **(i)** after the final clearing step using the clearing solvent BABB. All images were acquired with the same settings. For visualization purposes the magnification was kept at 40x in all images. The scale bar depicted in panel (**i**) represents 500 μm for all panels.

Upon deposition the samples were washed 2 times 15 minutes with PBS prior to overnight fixation with 4% paraformaldehyde (PFA, Merck, Darmstadt, Germany). Washing steps and incubation took place under agitation at 4 ⁰C unless stated otherwise. Subsequently the samples were cleared with a solvent based methodology [1], with slight modifications. First the samples were dehydrated via a graded methanol (MeOH) or alternatively by a graded ethanol (EtOH) series (Fisher Scientific, Waltham, Massachusetts, USA) starting at 25% and in incremental steps of 25% up to 100% solvent (either MeOH or EtOH). After dehydration, samples were taken up in a 1:1 (v/v) mixture of 100% MeOH or EtOH and a 2:1 (v/v) mixture of benzyl alcohol (Sigma Aldrich, Saint Louis, USA) and benzyl benzoate (Sigma Aldrich, Saint Louis, USA) resulting in a 50% MeOH or EtOH—50% BABB mixture. Finally the samples were taken up in 100% BABB, leading to complete clearing as shown in Fig 1. All incubation steps were carried out for 1 hour.

**Fig 2:**
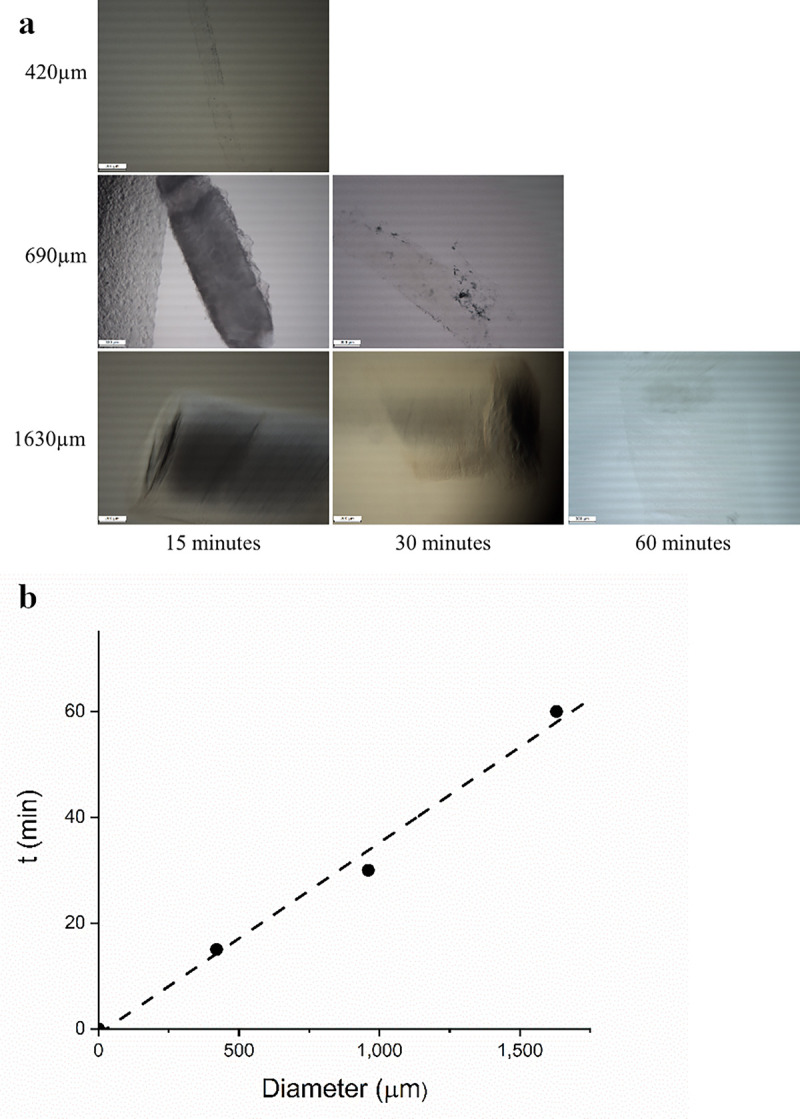
Determination of the minimum time needed for each process step to acquire optical transparent tissue. Shown in **(a)** a small tissue sample (D = 420 μm) that can be fully cleared by using 15-minute incubation steps and a 690 μm thick tissue needs 30-minute incubation steps to become fully optical transparant. Evaluating a tissue sample of approximately D = 1600 μm shows that at least 60-minute incubation steps are required to fully clear the tissue. A 300 μm scale bar is depicted in each image. The images contain horizontal stripes, this occurs due to the amount of transmitted light that goes directly into the sensor, leading to clipping. The graph **(b)** gives the incubation time needed to clear the tissue completely, plotted against the initial diameter, D.

Prior to this, in a series of experiments, the minimal processing time needed for complete clearing was investigated as a function of sample diameter *D*. It was found that the processing time needed depends on *D* in a linear fashion and clearing was complete when the processing steps lasted 1h (see Fig 2). The data in Fig 2 show that for 1 h processing time all samples up to a diameter D of 1600 μm were completely cleared. Hence, all incubation steps were carried out for 1 h, except for the ~ 3 mm thick samples, where the processing time was extended in accordance with extrapolation from Fig 2B.

Samples used in this study were obtained from redundant material and consisted of rat spleen and rat liver, while pig brain was obtained through a local butcher. Human prostate samples were commercially purchased (Proteogenex, Inglewood, California, USA). For all diameters, 5 independent samples for the rat tissue were taken, while from the human prostate tissue and pig brain 3 independent samples were taken. To follow the clearing procedure microscopic images were acquired after each incubation step with a microscope fitted with a camera (Leica, Wetzlar, Germany). To determine the shrinkage of the tissues during the clearing procedure, the diameters were determined using the scale bar function in Leica application suite (LAS) software. This was done at 6 different positions for each sample after each consecutive step in the clearing protocol.

### b. Measurement of shrinkage

**Fig 3:**
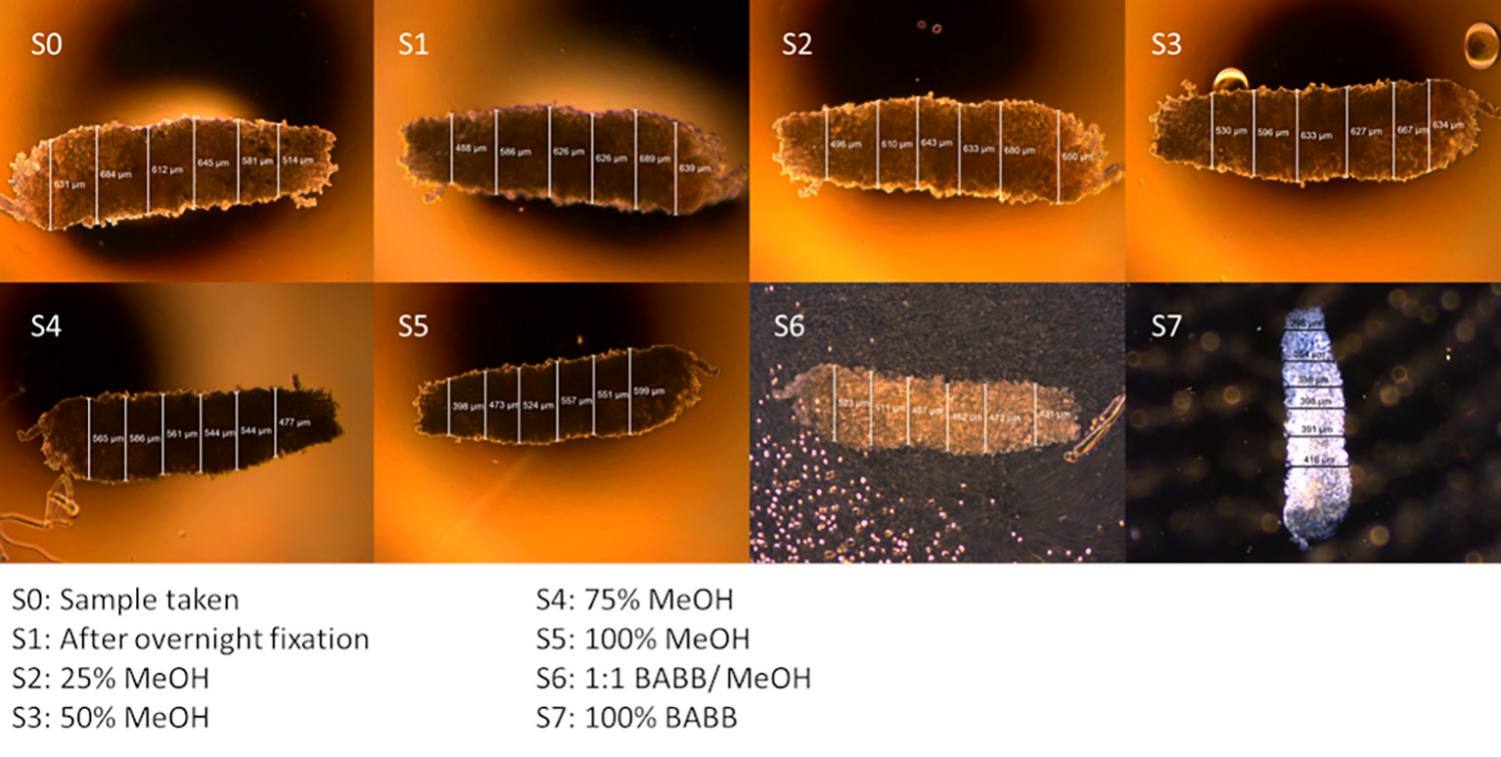
Measurement of tissue diameters during the 7 processing steps in the tissue clearing procedure. Shown is the measurement of the diameter of a single tissue sample, rat liver tissue in this particular example, for each of the processing steps (labelled Si). This figure shows the variation in diameter over the sample and the need to measure at several (in total 6) points, along the sample. The legend lists the solvents in which the tissue was incubated during the 7 process steps.

The tissue diameter was measured after the sample was taken from the biopsy needle, as we found that the needle diameter is not equal to the initial sample diameter. Moreover, this difference depends on tissue type. Subsequently, after each of the 7 processing steps in the clearing protocol, the sample diameters were measured. For each needle diameter, 5 samples were taken, while for human prostate and pig brain tissue 3 samples were taken for each needle diameter. At all stages, the diameter of each sample was measured at 6 points spread along the length of the entire sample as shown in Fig 3, using a scale bar available in the microscope (Leica application suite (LAS) software, version 090–135.001, Wetzlar, Germany). Thus a dispersion of measuring points and diameters was obtained, ensuring that the entire tissue is being measured for shrinkage and not just a particular part. The average of six measurements per sample was used as the sample diameter *D*.

## Results and discussion

**Fig 4:**
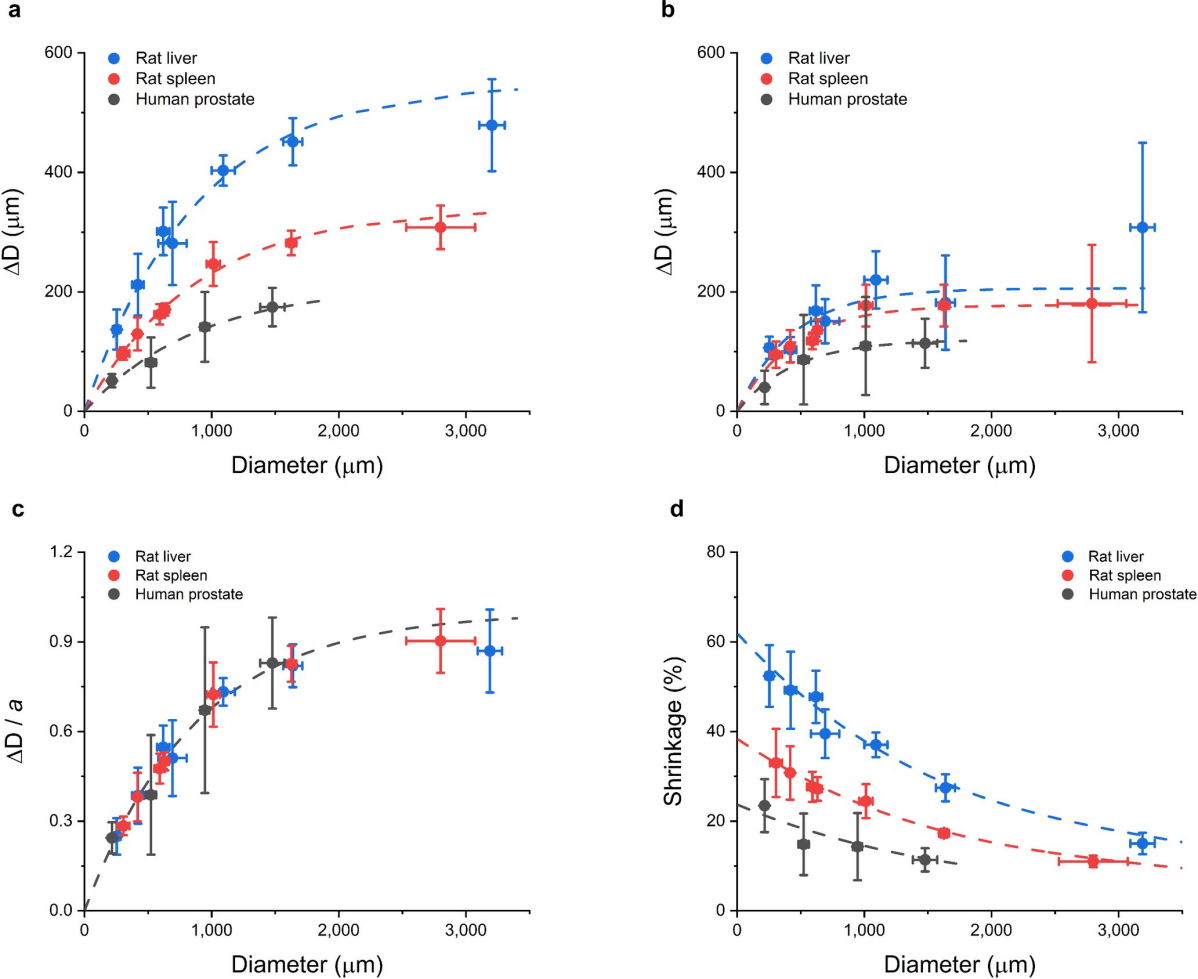
**(a)** Measured diameter change (ΔD) in μm for different tissues: Rat liver (blue), rat spleen (red) and human prostate (black) as a function of the initial sample thickness D due to sampling with different needle diameters. The dotted lines are a fits to the Eq (1) see text. **(b)** Measured diameter change (ΔD) in μm for the same three tissues as a function of the initial sample thickness D after the initial dehydration step. The dotted lines are fits to Eq (1) see text. Note that about half of the tissue shrinkage occurs in the initial dehydration step. **(c)** Diameter change from (a) normalized by the maximum change a as a function of the initial sample diameter. The line in the figure is a fit based on Eq (1), by virtue of the fact that the reference length L is the same for the three tissues. Note that the relative tissue shrinkage for all three tissue types falls on a universal curve. **(d)** The tissue shrinkage S = ΔD/D as a function of diameter for each of the three tissues examined. The dotted lines are based on Eq (2) using the fits obtained in (a). Note that the shrinkage for all three tissue types decreases with increasing D and thus is not a constant for a tissue.

The reduction in diameter, Δ*D*, for all used tissues and different initial diameters are given in Fig 4A. In Fig 4A the diameter change is plotted versus initial diameter for three different tissues; two of animal origin (rat liver and rat spleen) and one of human tissue origin (prostate). The reduction in diameter seems to level off with increasing initial tissue diameter. Note that after the procedure all samples were completely cleared, see Fig 1(i). To verify the levelling-off of the shrinkage for the thickest samples, the experiment for one of the thickest rat liver sample was repeated with the time of each processing steps extended from 1h to 3h. With Δ*D* = 407 ± 34 μm this did not yield a different result in the observed tissue shrinkage. To separate the effects of dehydration and clearing for the same three tissues, Fig 4B shows the diameter change during the initial dehydration stage only.

The trend in the diameter change can be captured by the function

ΔD=a[1−exp(−D/L)], (1)

which is of the type used in many branches of science to describe a gradual approach to saturation. In Eq (1), *a* is a tissue dependent factor giving the maximum shrinkage in the limit that the tissue diameter *D* → ∞, and *L* is a reference length. As shown in Fig 4A, this expression fits all three curves very well. A least-squares fit yields tissue dependent values *a* = 550 μm (liver), 340 μm (spleen) and 220 μm (prostate), respectively, while *L* = 880 μm for all three tissues. The dehydration data in Fig 4B can also be described by Eq (1), yet with fit values *a* = 206 μm (liver), 178 μm (spleen) and 120 μm (prostate), respectively, and the same value *L* = 435 μm for all three tissues. Not unexpectedly, the magnitude of the shrinkage depends on tissue type, consistent with recent literature reports [18]. Moreover, we note that the *a*-values, which reflect the tissue sensitivity to shrinkage, are in line with the general trend in fat percentage of these tissues [22]: that is, *a* increases with increasing fat content. This observation is consistent with the fact that both dehydration and BABB clearing include the removal of lipids [9]. Moreover, comparison of Fig 4A and 4B and the respective *a*-values reveals that approximately half the shrinkage occurs during the initial dehydration step with methanol (MeOH), while the other half occurs during the final tissue-clearing step in the MeOH-BABB tissue clearing process [1, 4, 17].

The observation in Fig 4A and 4B of only a change in the parameter *a* for different tissues immediately suggests that normalization of the diameter change by the tissue dependent factor *a* should give universal behavior, which is confirmed by Fig 4C.

The reduction in diameter of the samples is the direct read-out of the experiment, but is not a physically meaningful characteristic. The shrinkage *S* = Δ*D/D* is meaningful and is plotted in Fig 4D (in %, as is common in the literature). This figure shows clearly that the shrinkage, *S*, is *not* constant for a given tissue but depends on the sample diameter *D* in a manner which can be simply derived from Eq (1) to be:

S=(a/D)[1–exp(–D/L)]. (2)

The curves in Fig 4D are based on Eq (2) and the parameter values given above. A series expansion of the exponential in Eq (2) for small values of (*D*/*L*),

$$S\approx (a/L)–(a/L^{2})D,$$ (3)

reveals that the tissue shrinkage varies linearly with D for small samples and that *S* = *a/L* in the limit that *D*→0.

**Fig 5:**
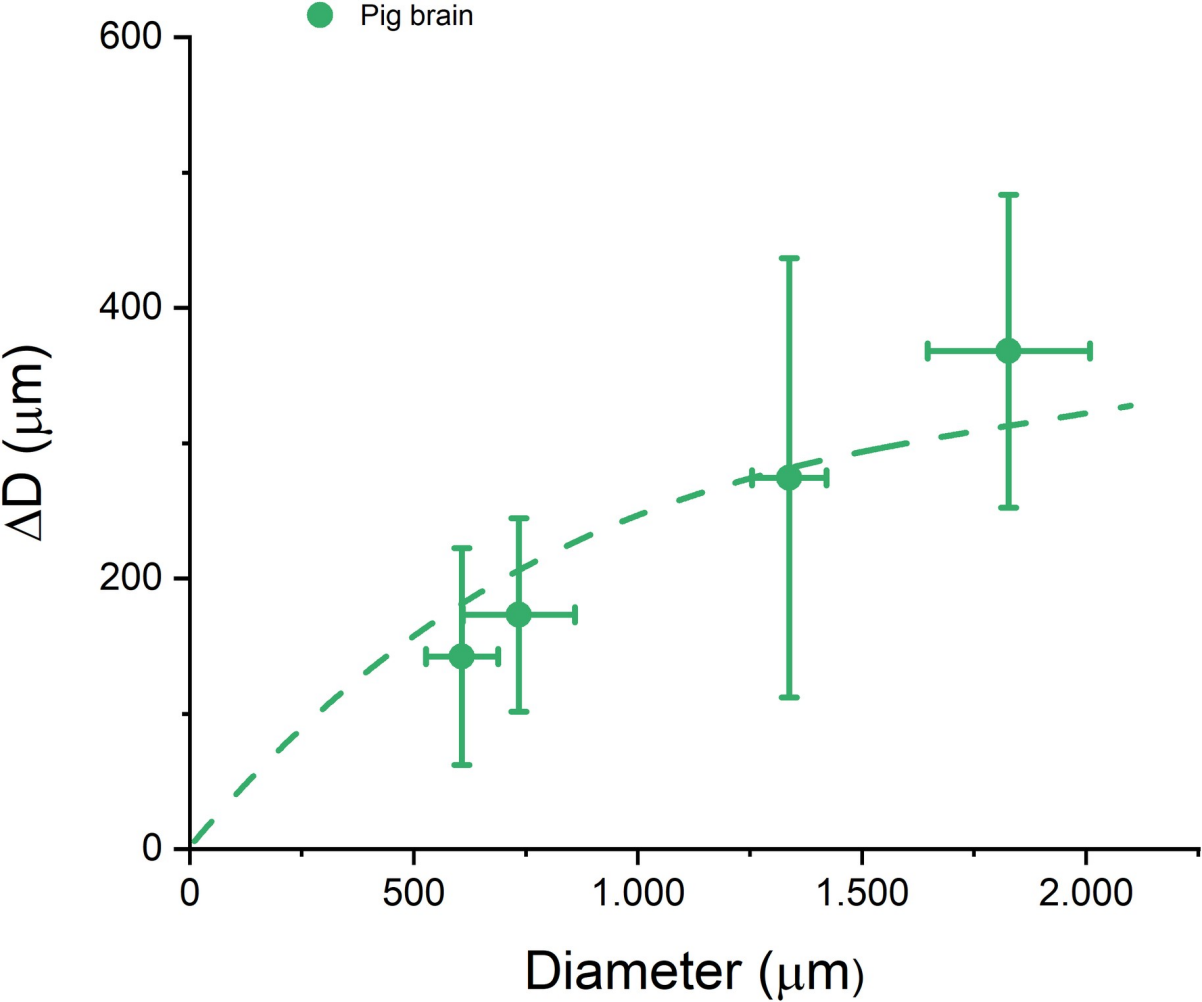
Diameter change (ΔD) in μm as function of diameter D for pig brain tissue. Again the shrinkage is seen to depend on D. The dashed curve is a fit to Eq (1), see text.

In the literature, also other dehydration reagents are being used, such as for instance ethanol (EtOH) [23] and tetrahydrofuran (THF) in the FDISCO method [14]. Therefore, we have investigated the effect of replacing methanol with ethanol on clearing-induced shrinkage for a 0.7 mm rat liver sample. When the dehydration series was performed using EtOH the shrinkage was 23.6 ± 6.3% (after dehydration) and 36.2 ± 10.8% (after BABB; cleared), while the use of MeOH gave a shrinkage of 23.8 ± 4.7% (after dehydration) and 40.0 ± 8.5% (after BABB; cleared). Within experimental accuracy, it thus appears that the shrinkage is the same for both dehydration agents. The tissues addressed above have an extracellular matrix (ECM) that primarily comprises collagen I [24, 25]. By contrast, the ECM of brain tissue is not protein based but rather almost entirely composed of glycosaminoglycans, such as hyaluronic acid [24, 26]. Fig 5 shows again a size dependence of the clearing-induced shrinkage in biopsy samples from pig brain. Despite the difference in ECM composition, the data reveal a similar trend as in Fig 4, with can be described by Eq (1) with parameters *a* = 360 μm and L = 880 μm. That this data shows more variation is partly caused by the difficulty of extracting smooth samples from this very soft tissue, and partly due to the unavoidable heterogeneity of brain tissue.

As recently noted, a detailed understanding of the physical and chemical principles underlying tissue-clearing processes is still missing, despite the significant advance in the efficacy of the tissues-clearing processes [11]. The experimental data presented here on shrinkage due to tissue clearing may help to shed light on these underlying mechanisms.

First of all, our experimental data present a caveat to the community that, in contrast to the existing literature, the amount of tissue shrinkage by BABB clearing depends on the tissue as well as on the size of the sample. Secondly, this work shows that it is possible to establish a closed-form empirical description of size dependent shrinkage, which will be instrumental for a quantitatively correct interpretation of images of BABB-cleared biopsies in the clinical practice [17, 19–21]. Thirdly, the observation that the reference length *L* is the same for the tissues investigated here, suggests that tissue shrinkage is governed the same physicochemical mechanism.

We emphasize that tissue shrinkage occurs in both the hydration phase and the clearing phase itself (see Fig 3). This would suggest that shrinkage is related to the de-lipidation, as this occurs in both stages of the clearing process [9]. Since, at least the solvent-based clearing methods have this same approach [9], one would expect that a similar shrinkrage dependence on sample size would be found for these methods.

At this point, we do not know the physical mechanism responsible for the observed size dependent clearing. Yet, it may be sought in the combination of the diffusion and reaction aspects of the clearing process itself. The experimental data presented here provides a detailed reference for the validation of a full microscopic and predictive model of the clearing process.

## Conclusion

We have shown that the tissue shrinkage using BABB clearing method is not fixed but depends on the sample size. For four different tissue types this dependence can be described by the empirical relation given by Eq (2), at least up to sample sizes of 3200 μm. Further work to examine this for other clearing agents should reveal how generally valid the results are.

## Supporting information

S1 Fig Preparation of solvent resistant containers. Depicted in (a) a dual barrel syringe container used to generate the solvent resistant container, (b) shows the assembling of the modified piston barrel onto a coverslip and (c) shows the completed container which can conveniently be used for imaging and solvent changes while minimizing manual handling of the samples.
(PDF)
