# Supplementary material for: Clearing-induced tisssue shrinkage: A novel observation of a thickness size effect
Source: PLoS One. 2021 Dec 16;16(12):e0261417. doi: 10.1371/journal.pone.0261417 (PMC8675714; doi:10.1371/journal.pone.0261417)
Supplement: S1 Fig — Depicted in (a) a dual barrel syringe container used to generate the solvent resistant container, (b) shows the assembling of the modified piston barrel onto a coverslip and (c) shows the completed container which can conveniently be used for imaging and solvent changes while minimizing manual handling of the samples. (PDF) [file pone.0261417.s001.pdf]

SUPPLEMENTARY FIGURES

a **b** **c**

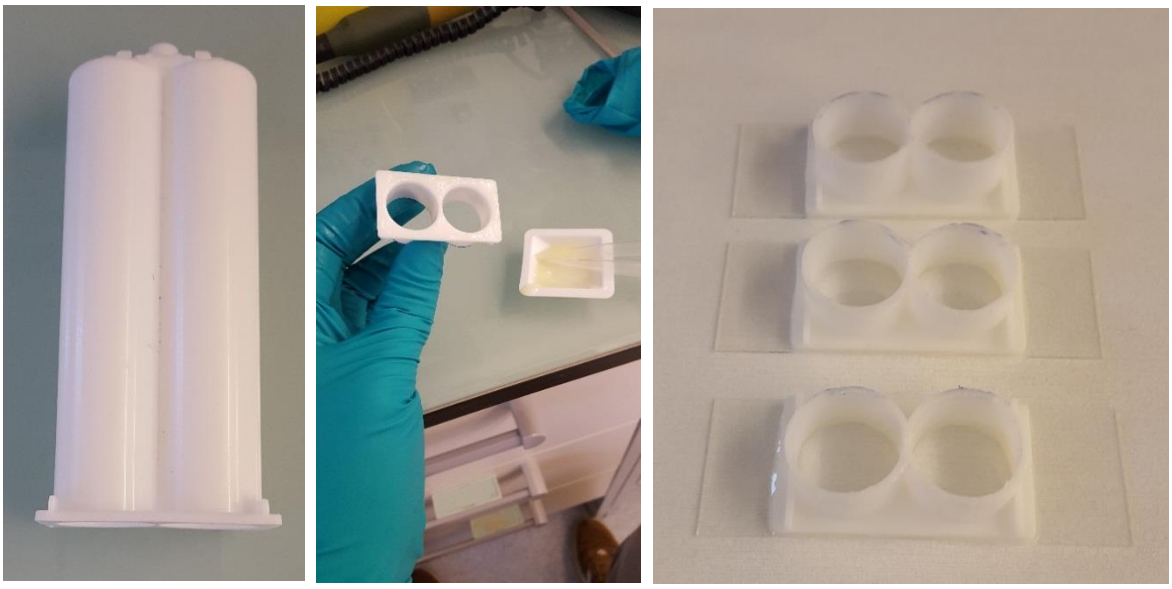

Supplementary Figure 1

Preparation of solvent resistant containers.

Depicted in **(a)** a dual barrel syringe container used to generate the solvent resistant container, **(b)** shows the assembling of the modified piston barrel onto a coverslip and **(c)** shows the completed container which can conveniently be used for imaging and solvent changes while minimizing manual handling of the samples.
